# Supplementary material for: ERCC1 and ERCC2 Polymorphisms Predict the Efficacy and Toxicity of Platinum-Based Chemotherapy in Small Cell Lung Cancer
Source: Pharmaceutics. 2024 Aug 25;16(9):1121. doi: 10.3390/pharmaceutics16091121 (PMC11434779; doi:10.3390/pharmaceutics16091121)
Supplement: Supplementary file 1 [file pharmaceutics-16-01121-s001.zip › pharmaceutics-3125154-supplementary.pdf]

Supplementary Table S1 Univariate associations between genetic variants and survival in the total cohort, limited-stage and extensive-stage subgroups

Total COHORT								
	Progression-free survival				Overall survival			
**SNP**	**n**	**mPFS (95%CI), months**	**HR (95% CI)**	***P*-value**	**n**	**mOS (95%CI), months**	**HR (95% CI)**	***P*-value**
ERCC1 rs11615								
AA	49	6.6 (5.8-7.4)	Reference (1)	0.06	50	11.3 (7.9-14.7)	Reference (1)	0.26
AG	52	7.0 (4.0-10.0)	0.66 (0.44-1.0)		53	11.8 (6.8-16.8)	0.72 (0.48-1.09)	
GG	16	7.7 (5.8-9.6)	0.57 (0.31-1.03)		18	12.7 (8.9-16.4)	0.75 (0.43-1.31)	
AG-GG[a]	68	7.4 (5.6-9.1)	0.64 (0.43-0.94)	**0.02**	71	12.7 (8.6-16.7)	0.73 (0.50-1.07)	0.1
ERCC1 rs3212986								
GG	70	7.1 (6.2-8.0)		0.71	73	14.6 (10.8-18.3)		0.88
GA	42	6.4 (5.5-7.2)			43	9.2 (7.0-11.4)		
AA	5	10.2 (4.8-15.5)			5	14.2 (10.3-18.0)		
ERCC2 rs13181								
TT	52	7.3 (6.5-8.2)		0.91	54	13.2 (8.1-18.3)		0.69
TG	53	6.4 (4.7-8.0)			54	11.2 (5.9-16.6)		
GG	12	6.3 (5.1-7.4)			13	11.5 (7.1-16.0)		
ERCC2 rs50872								
GG	60	7.3 (6.4-8.3)		0.90	63	14.6 (9.7-19.4)		0.74
GA	50	6.1 (5.5-6.6)			51	10.4 (8.3-12.5)		
AA	7	7.8 (6.1-9.6)			7	17.0 (12.5-21.6)		
ERCC2 rs1799793								
CC	52	7.3 (5.9-8.8)		0.70	53	14.9 (11.8-17.9)		0.38
CT	53	6.4 (4.9-7.8)			55	11.2 (6.6-15.9)		
TT	12	6.0 (4.2-7.8)			13	10.4 (7.4-13.4)		
XRCC1 rs25487								

CC	40	7.0 (6.1-7.8)		0.56	41	14.9 (9.2-20.5)		0.66
CT	62	7.1 (5.9-8.3)			64	11.8 (8.8-14.9)		
TT	15	6.5 (5.7-7.3)			16	10.4 (8.5-12.3)		
ABCC3 rs4793665								
TT	35	6.4 (5.4-7.4)	Reference (1)	0.18	35	10.1 (7.5-12.7)	Reference (1)	**0.046**
TC	59	7.7 (5.7-9.8)	0.67 (0.43-1.03)		62	14.6 (10.4-18.7)	0.59 (0.38-0.90)	
CC	23	7.3 (6.1-8.6)	0.80 (0.47-1.34)		24	12.7 (7.3-18.0)	0.77 (0.46-1.31)	
ABCB1 rs1045642								
GG	35	6.4 (5.9-7.0)		0.51	36	12.7 (8.8-16.5)		0.99
GA	54	7.3 (5.6-9.1)			57	10.4 (7.0-13.8)		
AA	28	6.9 (5.8-8.2)			28	15.5 (8.1-23.0)		
ABCB1 rs2032582								
GG	39	6.4 (5.8-7.0)		0.45	40	12.7 (6.8-18.5)		0.96
GT-GA	55	7.3 (5.7-8.9)			58	10.6 (7.2-13.9)		
TT-AT	23	7.1 (5.9-8.3)			23	15.7 (6.6-24.7)		
ABCB1 rs1128503								
GG	36	6.1 (5.1-7.2)		0.63	37	11.5 (7.1-16.0)		0.35
GA	55	7.3 (5.5-9.2)			58	11.2 (7.7-14.8)		
AA	26	7.4 (6.3-8.4)			26	15.7 (8.0-23.3)		
UGT1A1 rs3064744								
*1/*1	57	7.3 (6.0-8.7)		0.15	58	13.2 (8.9-17.4)		0.31
*1/*28	41	6.6 (6.0-7.2)			43	11.3 (9.0-13.5)		
*28/*28	19	8.4 (4.7-12.0)			19	13.8 (8.5-19.1)		
GSTP1 rs1695								
AA	55	4.1 (6.3-7.9)		0.63	56	11.2 (7.8-14.7)		0.28
AG	46	6.6 (5.3-7.9)			48	13.8 (8.3-19.3)		
GG	16	6.5 (5.1-7.8)			17	11.5 (5.0-18.0)		

	LIMITED-STAGE							
	Progression-free survival				Overall survival			
**SNP**	**n**	**mPFS (95%CI), months**	**HR (95% CI)**	***P*-value**	**n**	**mOS (95%CI), months**	**HR (95% CI)**	***P*-value**
ERCC1 rs11615								
AA	15	10.8 (8.0-13.6)	Reference (1)	**0.009**	15	20.7 (14.3-27.1)	Reference (1)	**0.04**
AG	17	18.2 (10.2-26.2)	0.3 (0.13-0.68)		18	33.8 (26.8-40.7)	0.38 (0.17-0.83)	
GG	10	9.6 (0.0-28.5)	0.43 (0.17-1.1)		10	14.8 (0.0-45.4)	0.73 (0.32-1.7)	
AG-GG[a]	27	18.2 (8.1-28.3)	0.34 (0.16-0.71)	**0.003**	28	33.8 (22.2-45.4)	0.48 (0.24-0.96)	**0.03**
ERCC1 rs3212986								
GG	24	10.8 (8.5-13.1)		0.22	24	23.0 (13.6-32.5)		0.72
GA	15	18.2 (8.0-28.4)			16	25.8 (0.0-62.9)		
AA	3	20.6 (0.0-43.2)			3	34.8 (0,0-79.3)		
ERCC2 rs13181								
TT	18	12.2 (7.0-17.3)		0.49	19	25.8 (15.7-35.9)		0.32
TG	18	12.5 (5.1-19.8)			18	33.7 (9.2-58.3)		
GG	6	8.4 (.8-15.9)			6	14.8 (7.8-21.9)		
ERCC2 rs50872								
GG	23	13.3 (9.3-17.4)	Reference (1)	**0.03**	24	31.8 (19.4-44.3)	Reference (1)	0.16
GA	14	14.9 (5.5-24.3)	0.66 (0.3-1.45)		14	16.1 (0.0-44.6)	1.00 (0.47-2.13)	
AA	5	7.8 (6.4-9.3)	2.9 (1.03-8.17)		5	15.3 (6.7-23.9)	2.55 (0.92-7.09)	
GG-GA[b]	37	14.8 (10.6-18.9)	0.30 (0.11-0.82)	**0.01**	38	28.7 (16.7-40.7)	0.39 (0.15-1.06)	0.06
ERCC2 rs1799793								
CC	19	12.2 (6.5-17.8)		0.78	19	28.7 (16.2-41.2)		0.57
CT	19	12.6 (9.6-15.5)			20	20.7 (0.2-41.2)		
TT	4	8.4 (0.0-23.0)			4	9.4 (0.0-34.8)		
XRCC1 rs25487								

CC	14	13.3 (5.9-20.8)		0.75	14	25.5 (11.7-39.3)		0.78
CT	21	12.2 (9.6-14.8)			21	31.8 (17.0-46.7)		
TT	7	12.5 (1.9-23.0)			18	9.4 (0.0-25.7)		
ABCC3 rs4793665								
TT	9	11.2 (9.6-12.8)	Reference (1)	0.24	9	23.0 (0.0-55.8)	Reference (1)	**0.04**
TC	25	14.8 (10.8-18.7)	0.57 (0.26-1.29)		25	35.2 (32.8-37.5)	0.40 (0.17-0.92)	
CC	8	9.6 (4.5-14.7)	1.05 (0.39-2.84)		9	19.0 (7.0-30.9)	0.96 (0.38-2.51)	
ABCB1 rs1045642								
GG	15	11.2 (8.0-14.4)		0.25	15	21.1 (7.4-34.9)		0.90
GA	18	14.9 (10.7-19.1)			19	33.7 (22.8-44.6)		
AA	9	10.7 (6.1-15.3)			9	23.0 (16.2-29.9)		
ABCB1 rs2032582								
GG	16	11.2 (8.6-13.8)		0.36	16	25.8 (0.5-51.0)		0.70
GT_GA	19	14.9 (10.9-18.9)			20	25.5 (9.8-41.1)		
TT_AT	7	10.7 (6.6-14.7)			7	23.0 (17.0-29.1)		
ABCB1 rs1128503								
GG	12	10.8 (6.2-15.4)		0.88	12	21.2 (0.0-46.3)		0.16
GA	20	14.9 (10.5-19.3)			21	25.8 (8.0-43.6)		
AA	10	10.7 (5.4-15.9)			10	23.0 (0.6-45.5)		
*UGT1A1*28* rs3064744								
*1/*1	17	19.1 (15.1-23.1)	Reference (1)	0.05	17	34.8 (29.1-40.6)	Reference (1)	0.23
*1/*28	15	10.8 (6.6-15.1)	2.57 (1.17-5.64)		16	15.3 (7.2-23.3)	1.90 (0.89-4.07)	
*28/*28	10	9.1 (5.7-12.5)	1.84 (0.75-4.54)		10	16.1 (0.0-45.4)	1.17 (0.48-2.86)	
*1/*28-*28/*28[a]	25	10.8 (8.0-13.6)	2.24 (1.1-4.57)	**0.02**	26	16.1 (8.8-23.4)	1.559 (0.78-3.10)	0.2
GSTP1 rs1695								
AA	19	10.8 (8.1-13.6)		0.36	19	19.0 (3.4-34.5)		0.30
AG	17	14.8 (11.5-18.0)			18	31.8 (13.3-50.3)		
GG	6	10.7 (5.9-15.4)			6	23.1 (0.0-46.5)		

	EXTENSIVE-STAGE							
	Progression-free survival				Overall survival			
**SNP**	**n**	**mPFS (95%CI), months**	**HR (95% CI)**	***P*-value**	**n**	**mOS (95%CI), months**	**HR (95% CI)**	***P*-value**
ERCC1 rs11615								
AA	34	5.9 (4.9-6.9)		0.97	35	10.3 (9.3-11.3)		0.97
AG	35	6.1 (5.0-7.2)			35	8.4 (7.1-9.8)		
GG	6	4.6 (0.5-8.7)			8	5.6 (0.0-15.5)		
ERCC1 rs3212986								
GG	46	6.0 (4.8-7.2)	Reference (1)	**0.049**	49	10.6 (8.6-12.5)	Reference (1)	0.1
GA	27	5.4 (3.8-6.9)	1.81 (1.08-3.00)		27	7.5 (5.8-9.2)	1.69 (1.04-2.73)	
AA	2	7.7 (NA-NA)	0.67 (0.16-2.75)		2	12.4 (NA-NA)	1.06 (0.26-4.42)	
ERCC2 rs13181								
TT	34	6.5 (5.9-7.0)		0.5	35	9.8 (8.2-11.4)		0.78
TG	35	4.9 (3.9-5.8)			36	8.1 (6.6-9.7)		
GG	6	4.9 (3.2-6.6)			7	11.5 (8.6-14.4)		
ERCC2 rs50872								
GG	37	6.3 (5.9-6.8)		0.81	39	9.8 (7.1-12.5)		0.78
GA	36	5.3 (3.9-6.7)			37	9.1 (6.8-11.4)		
AA	2	2.2 (NA-NA)			2	17.0 (NA-NA)		
ERCC2 rs1799793								0.49
CC	33	6.5 (5.9-7.0)		0.44	34	9.8 (8.3-11.3)		0.51
CT	34	4.9 (3.6-6.1)			35	8.4 (5.7-11.1)		
TT	8	4.9 (3.2-6.6)			9	10.4 (1.9-18.8)		
XRCC1 rs25487								
CC	26	6.1 (5.2-7.1)		0.19	27	9.7 (6.4-12.9)		0.31
CT	41	6.1 (5.2-6.9)			43	9.2 (6.7-11.7)		
TT	8	4.4 (3.2-5.6)			8	10.4 (5.4-15.4)		

ABCC3 rs4793665						
TT	26	4.9 (3.3-6.4)	0.50	26	7.4 (5.4-9.5)	0.67
TC	34	5.5 (4.7-6.3)		37	10.3 (8.3-12.3)	
CC	15	6.6 (5.3-7.8)		15	10.4 (8.1-12.7)	
ABCB1 rs1045642						
GG	20	4.9 (3.8-5.9)	0.18	21	8.1 (3.8-12.4)	0.35
GA	36	6.0 (4.7-7.4)		38	8.4 (6.4-10.4)	
AA	19	6.5 (5.7-7.2)		19	13.8 (6.5-21.2)	
ABCB1 rs2032582						
GG	23	5.4 (4.9-5.8)	0.13	24	8.0 (5.9-10.1)	0.19
GT_GA	36	6.1 (4.7-7.4)		38	9.1 (6.8-11.4)	
TT_AT	16	6.5 (6.0-6-9)		16	11.2 (1.5-21.0)	
ABCB1 rs1128503						
GG	24	5.4 (4.9-5.8)	0.13	25	8.2 (6.8-9.6)	0.14
GA	35	6.0 (5.0-7.1)		37	9.2 (6.9-11.5)	
AA	16	6.6 (5.6-7.6)		16	11.5 (1.8-21.2)	
UGT1A1 rs3064744						
*1/*1	40	6.1 (5.1-7.1)	0.59	41	10.4 (8.3-12.5)	0.83
*1/*28	26	6.0 (4.1-7.9)		27	8.2 (6.3-10.0)	
*28/*28	9	5.4 (5.1-5.7)		9	10.1 (9.2-11.0)	
GSTP1 rs1695						
AA	36	6.5 (5.7-7.3)	0.74	37	10.3 (8.8-11.8)	0.58
AG	29	5.2 (4.3-6.2)		30	8.2 (4.6-11.7)	
GG	10	6.1 (4.9-7.3)		11	9.8 (6.8-12.9)	

[a] Dominant model; [b] Recessive model; * Fisher test

Statistically significant *P*-values are marked in bold.

Supplementary Table S2 Univariate associations between genetic variants and objective response rate (ORR) in the total cohort, limited-stage and extensive-stage subgroups

	OBJECTIVE RESPONSE RATE<br>Chemotherapy* (n=94)		
**SNP**	**Affected, n/ Total, n**	**%**	***P*-value**
ERCC1 rs11615			
AA	34/41	82.9	0.92
AG	36/42	85.7	
GG	9/11	81.8	
ERCC1 rs3212986			
GG	47/56	83.9	0.74
GA	29/35	82.9	
AA	2/3	66.7	
ERCC2 rs13181			
TT	35/40	87.5	0.22
TG	36/46	78.3	
GG	8/8	100	
ERCC2 rs50872			
GG	36/45	80	0.45
GA	40/45	88.9	
AA	3/4	75	
ERCC2 rs1799793			
CC	35/41	85.4	0.76
CT	35/43	81.4	
TT	9/10	90	
XRCC1 rs25487			
CC	25/29	86.2	0.97
CT	43/51	84.3	
TT	12/14	85.7	
ABCC3 rs4793665			
TT	25/31	80.6	0.12
TC	36/45	80	
CC	18/18	100	
ABCB1 rs1045642			
GG	22/26	84.6	0.54
GA	37/46	80.4	
AA	20/22	90.9	
ABCB1 rs2032582			
GG	23/30	76.7	0.36
GT-GA	40/45	88.9	
TT-AT	16/19	84.2	
ABCB1 rs1128503			
GG	22/29	75.9	0.32
GA	38/44	86.4	
AA	19/21	76.2	

UGT1A1 rs3064744			
*1/*1	36/46	78.3	0.29
*1/*28	29/33	87.9	
*28/*28	14/15	93.3	
GSTP1 rs1695			
AA	40/46	87	0.73
AG	29/36	80.6	
GG	10/12	83.3	

*Evaluated in patients undergoing chemotherapy or sequential chemo-radiotherapy

Supplementary Table S3 Univariate associations between genetic variants and chemotherapy-related toxicities in the total cohort

	ANAEMIA			THROMBOCYTOPENIA			NEUTROPENIA		
**SNP**	**Affected, n/ Total, n**	**(%)**	***P*-value**	**Affected, n/ Total, n**	**(%)**	***P*-value**	**Affected, n/ Total, n**	**(%)**	***P*-value**
XRCC1 rs25487									
CC	9/52	17.3	0.12	11/52	21.2	0.65	34/52	65.4	0.85
CT	15/71	21.1		12/71	16.9		43/70	61.4	
TT	0/17	0.0		2/17	11.8		10/17	58.8	
CC-CT[b]	24/123	19.5	**0.04***						
ERCC1 rs11615									
AA	11/57	19.3	0.80	10/57	17.5	1.00	34/57	59.6	0.69
AG	9/61	14.8		11/61	18.0		40/60	66.7	
GG	4/22	18.2		4/22	18.0		13/22	59.1	
ERCC1 rs3212986									
GG	15/81	18.5	0.88	13/81	16.0	0.78	52/81	64.2	0.32
GA	8/53	15.1		11/53	20.8		33/52	63.5	
AA	1/6	16.7		1/6	16.7		2/6	33.0	
ERCC2 rs50872									
GG	11/74	14.9	**0.04**	14/74	18.9	0.76	50/73	68.5	0.16
GA	9/58	15.5		9/58	15.5		31/58	53.4	
AA	4/8	50.0		2/8	25.0		6/8	75.0	
GG-GA[b]	20/132	15.2	**0.03***						
ERCC2 rs1799793									
CC	10/61	16.4	0.91	6/61	9.8	**0.04**	40/61	65.6	0.27
CT	12/65	18.5		14/65	21.5		41/64	64.1	
TT	2/14	14.3		5/14	35.7		6/14	42.9	

CT-TT[a]				19/79	24.1	**0.03**			
ERCC2 rs13181									
TT	13/59	22.0	0.42	7/59	11.9	0.29	37/59	62.7	0.57
TG	9/67	13.4		15/67	22.4		43/66	65.2	
GG	2/14	14.3		3/14	21.4		7/14	50.0	
ABCC3 rs4793665									
TT	6/41	14.6	0.85	9/41	22.0	0.71	24/40	60.0	0.88
TC	13/71	18.3		12/71	16.9		46/71	64.8	
CC	4/27	14.8		4/27	14.8		17/27	63.0	
ABCB1 rs1045642									
GG	7/44	15.9	0.14	7/44	15.9	0.56	28/43	65.1	0.28
GA	7/62	11.3		10/62	16.1		35/62	56.5	
AA	9/33	27.3		8/33	24.2		24/33	72.7	
ABCB1 rs2032582									
GG	10/52	19.2	0.63	9/52	17.3	0.88	34/51	66.7	0.25
GT-GA	8/61	13.1		12/61	19.7		34/61	55.7	
TT-AT	5/26	19.2		4/26	15.4		19/26	73.1	
ABCB1 rs1128503									
GG	9/50	18.0	0.91	8/50	16.0	0.87	31/49	63.3	0.44
GA	9/60	15.0		11/60	18.3		35/60	58.3	
AA	5/29	17.2		6/29	20.7		21/29	72.4	
UGT1A1 rs3064744									
*1/*1	10/64	15.6	0.36	12/64	18.8	0.05	40/64	62.5	0.97
*1/*28	6/50	12.0		5/50	10.0		32/50	64.0	
*28/*28	6/24	25.0		8/21	33.3		15/23	65.2	
*1/*1-*1/*28[b]				17/114	14.9	**0.03**			
GSTP1 rs1695									
AA	10/61	16.4	1.00	9/61	14.8	0.67	40/60	66.7	0.60

AG	10/60	16.7	12/60	20.0	35/60	58.3
GG	3/18	16.7	4/18	22.2	12/18	66.7

[a] Dominant model; [b] Recessive model; * Fisher test

Statistically significant *P*-values are marked in bold.

Supplementary Table S4 Univariate associations between genetic variants and radiotherapy-related esophagitis in limited-stage

	ESOPHAGITIS		
**SNP**	**Affected, n/Total, n**	**(%)**	***P*-value**
XRCC1 rs25487			
CC	4/10	40.0	0.80
CT	6/18	33.3	
TT	2/8	25.0	
ERCC1 rs11615			
AA	3/11	27.3	0.52
AG	5/17	29.4	
GG	4/8	50.0	
ERCC1 rs3212986			
GG	5/19	26.3	0.38
GA	5/14	35.7	
AA	2/3	66.7	
ERCC2 rs50872			
GG	8/19	42.1	0.06
GA	1/12	8.3	
AA	3/5	60.0	
ERCC2 rs1799793			
CC	6/14	42.9	0.05
CT	3/18	16.7	
TT	3/4	75.0	
ERCC2 rs13181			
TT	5/14	35.7	0.10
TG	3/16	18.8	
GG	4/6	66.7	
